# Impact of non-LTR retrotransposons in the differentiation and evolution of anatomically modern humans

**DOI:** 10.1186/s13100-018-0133-4

**Published:** 2018-08-15

**Authors:** Etienne Guichard, Valentina Peona, Guidantonio Malagoli Tagliazucchi, Lucia Abitante, Evelyn Jagoda, Margherita Musella, Marco Ricci, Alejandro Rubio-Roldán, Stefania Sarno, Donata Luiselli, Davide Pettener, Cristian Taccioli, Luca Pagani, Jose Luis Garcia-Perez, Alessio Boattini

**Affiliations:** 1 Department of Biological, Geological and Environmental Sciences, University of Bologna, 40126 Bologna, Italy; 2 Department of Evolutionary Biology (EBC), Uppsala University, SE-752 36 Uppsala, Sweden; 3 Department of Research and Innovation, Azienda Ospedaliero Universitaria di Parma, 43126 Parma, Italy; 4 Human Evolutionary Biology, Harvard University, Cambridge, MA 02138 USA; 5 GENYO - Pfizer - Universidad de Granada - Junta de Andalucía Centre for Genomics and Oncological Research, PTS Granada, 18007 Granada, Spain; 6 Department of Cultural Heritage, University of Bologna, Ravenna Campus, 48121 Ravenna, Italy; 7 Department of Animal Medicine, Production and Health, University of Padova, 35020 Legnaro, Pd Italy; 8 Department of Biology, University of Padova, 35131 Padova, Italy; 9 Estonian Biocentre, Institute of Genomics, University of Tartu, 51010 Tartu, Estonia; 10 MRC Human Genetics Unit, Institute of Genetics and Molecular Medicine (IGMM), University of Edinburgh, Western General Hospital, Edinburgh, EH4 2XU UK

**Keywords:** Non-LTR retrotranspososons, Human evolution, Ancient genomes, Chimpanzees, Generation of variability, Functional analyses

## Abstract

**Background:**

Transposable elements are biologically important components of eukaryote genomes. In particular, non-LTR retrotransposons (N-LTRrs) played a key role in shaping the human genome throughout evolution. In this study, we compared retrotransposon insertions differentially present in the genomes of Anatomically Modern Humans, Neanderthals, Denisovans and Chimpanzees, in order to assess the possible impact of retrotransposition in the differentiation of the human lineage.

**Results:**

We first identified species-specific N-LTRrs and established their distribution in present day human populations. These analyses shortlisted a group of N-LTRr insertions that were found exclusively in Anatomically Modern Humans. These insertions are associated with an increase in the number of transcriptional/splicing variants of those genes they inserted in. The analysis of the functionality of genes containing human-specific N-LTRr insertions reflects changes that occurred during human evolution. In particular, the expression of genes containing the most recent N-LTRr insertions is enriched in the brain, especially in undifferentiated neurons, and these genes associate in networks related to neuron maturation and migration. Additionally, we identified candidate N-LTRr insertions that have likely produced new functional variants exclusive to modern humans, whose genomic loci show traces of positive selection.

**Conclusions:**

Our results strongly suggest that N-LTRr impacted our differentiation as a species, most likely inducing an increase in neural complexity, and have been a constant source of genomic variability all throughout the evolution of the human lineage.

**Electronic supplementary material:**

The online version of this article (10.1186/s13100-018-0133-4) contains supplementary material, which is available to authorized users.

## Background

Transposable Elements (TEs) are DNA sequences that are able to move or replicate in genomes via cut-and-paste and copy-and-paste-like mechanisms [1]. Although TEs have for long been dismissed as “selfish”, “parasites” or “junk DNA” [2, 3], the advent of whole genome DNA sequencing, in conjunction with molecular genetic, biochemical, genomic and functional studies, has revealed that TEs are biologically important components of mammalian genomes whose activity significantly influenced the structure and function of our own genome [1]. TEs are known to be involved in several evolutionary and adaptive processes such as the generation of genes and pseudogenes [4–6], fine-tuning transcriptional regulation of genes [7–9], generation of somatic mosaicism [10–12], the increase in complexity and evolution of gene regulatory networks [13] and the alteration of epigenetic mechanisms as processes of fine-scale and reversible regulation [14]. Some of the most notable biological processes associated with the domestication of TE-derived sequences are the insurgence of the V (D) J system of acquired immunity [15–17] and placental development [18, 19], but they also play key roles in embryogenesis [20–22] and possibly neurogenesis [11, 12, 23]. In sum, in addition to their role in growing the size of eukaryotes’ genomes [14] through generation of new copies of themselves, active TEs are continually impacting the functioning and evolution of genomes.

Notably, the activity of TEs throughout evolution has generated roughly half of the human genomic material [24, 25]. In modern humans, only a limited number of TE subfamilies from the non-LTR-retrotransposon (N-LTRr) class are currently active, i.e. LINE-1 s, Alus and SVAs. Indeed, the ongoing activity of these N-LTRrs in humans offers a constant source of inter-individual variability in human populations and can sporadically generate new genetic disorders [1, 26].

Thanks to the recent development of Next-Generation sequencing techniques and to the advances in the ancient DNA field, a large number of genomes from the human and chimpanzee lineage are currently available, including not only several modern human populations, but also our most recent extinct relatives, i.e. Neanderthals and Denisovans.

*Homo neanderthalensis* (HN) and Denisovan hominids (HD) are sibling clades, being more closely related to each other than they are to *Homo sapiens*. Their split from the modern human lineage is estimated to have occurred between 550 thousands of years ago (Kya) and 765 Kya, after which they colonized Eurasia long before Anatomically Modern Humans (AMH) left Africa. The population split between these archaic populations is estimated at 381–473 kya [27]. Notably, the genomes of some individuals belonging to HN and HD have been previously sequenced, assembled and published [27–30], two of which are high-coverage: one HD and one HN, both from the Altai mountains in southern Siberia [27, 29].

Such unprecedented amount of genomic data allowed thorough investigations of the genomic changes occurred along the human lineage, in some cased leading to the identification of variants that had a potential role in the evolution of our species [31]. However, these studies focused almost exclusively on point mutations such as SNPs and short InDels.

Although often discussed and speculated about, the effects and implications of Retrotransposon Insertions (RIs) on the evolution of the human lineage are mostly unknown. Indeed, other studies already identified a repertoire of RIs differentially present in the genomes of AMH, HN, HD and chimpanzees [32], but the functional impact of RIs and their evolutionary role is still unexplored. Therefore, with this study we aim at evaluating the potential impact of RIs in AMH differentiation and evolution in comparison to our closest relatives: i) we characterize the locations of inheritable RIs that are exclusive to our species, as well as to HN, HD and chimps, ii) identify potential selective pressures and iii) infer functional/regulatory alterations that might have occurred in specific tissues after their insertion.

## Results

### RIs identification

Available RI identification tools such as RetroSeq [33], Tangram [34], TEA [35], MELT [32], etc. are primarily based on mapping paired-end DNA-sequencing reads. However, and given that a large portion of previously sequenced ancient DNAs is composed of single-ended reads, here we devised a methodology for detecting differentially present RIs in AMH, HD and HN genomes based on single-ended reads (Additional file 1). In particular, our methodology is meant to identify species-specific RIs by locating 3′ ends of insertions differentially present in two genomes, then confirming their 5′ ends and absence from the other species’ genome (details in METHODS).

Since few high-coverage ancient genomes are currently available, the absolute specificity of RIs cannot be ascertained. Therefore, in this study we intend the term “species-specific” as in relation to the compared species’ genome.

Our analyses led to the identification of: i) 507 putative HN-specific and 331 putative HD-specific RIs, and ii) 3215 and 7185 putative AMH-specific RIs vs HN and HD, respectively.

As for the comparison between AMH (GRCh37-hg19) and chimpanzee (panTro5) genomes, we retrieved all RIs annotated in RepBase [36] in these two genomes and analyzed the presence/absence of the insertions in the reference sequences of the two species.

Next, we developed a computational validation procedure, through which we eliminated all those insertions that presented uncertainties in mobile element subfamily attribution or whose location might be ambiguous (see METHODS for details). Thus, we only continued the analysis of the most reliable and canonically-inserted RIs identified (Additional file 1).

A number of species-specific RI were computationally validated: 1917 Chimp-specific, 38 HD-specific, 64 HN-specific, 5402 AMH-specific (against chimps), 548 AMH-specific (against Denisova) and 806 AMH-specific (against Neanderthal) (Table 1, Additional files 2, 3, 4, 5, 6, 7, 8). The validation method thus excluded approximately 87% of the identified insertions, which could have presented any sort of bias or uncertainty in attribution. Of the validated AMH-specific RIs, 321 were present in the modern human genome and were absent in both HN and HD genomes.

**Table 1:** Number of identified and validated RIs in chimpanzee, HN, HD and AMH genomes

	TOTAL	Alu	LINE-1	SVA
Chimp-specific RIs	1917	1170	614	133
HN-specific RIs	64	57	6	1
HD-specific RIs	38	32	6	0
AMH-specific RIs vs. chimp	5402	4504	655	253
AMH-specific RIs vs. HN	806	728	77	1
AMH-specific RIs vs. HD	548	487	58	3
AMH-specific RIs vs. both HN and HD	321	295	25	1

A large dataset (defined as RetroTransposon DataBase - RT-DB) of ~ 666,000 reference retrotransposon insertions from the most recent subfamilies of N-LTRrs (i.e. AluS, AluY, LINE-1HS, LINE-1PA2, LINE-1PA3, LINE-1PA4 and all SVAs) present in the reference GRCh37-hg19 was retrieved in order to assess if characteristics of loci containing AMH-specific insertions were random, retrotransposition-dependent or peculiar to the human lineage. The comparison of the identified insertions with RT-DB ones revealed that the activity of N-LTRrs in the human lineage has remained consistent: Alu RIs are far more common than LINE-1 RIs, while SVAs produced only a handful of insertions.

According to our results, the patterns of retrotransposition in the human lineage seem to have remained relatively constant (0.6–0.8 insertions/Ky), while the rate of RI accumulation in chimps (0.29 insertions/Ky) has been approximately 2.5 times lower than in humans.

### Archaic-specific RIs and insertional polymorphisms

HD- and HN-specific RIs (38 and 64, respectively) were compared between the two species and with present-day AMH populations data from 1000 Genomes Project Phase 3 (Additional files 9A-B, 10, 11) [37]. All abbreviations used in this paper for modern human populations names follow the definition and usage by the 1000 Genomes Project [37]. Based on the available 1000 Genomes Project data, three RIs were found in both archaic species, while nearly half of them (49 out of 102) are polymorphic to various degrees in modern populations. Interestingly, 8 of these insertions (1 HD-specific and 7 HN-specific) are absent in African (AFR) individuals and present at a low frequency only in some (or all) non-AFR populations, thus mirroring the well-known admixture patterns between modern humans and archaic species [27, 28, 32] .

Since some putative archaic-specific RIs are polymorphic in modern humans, it is likely that at least some putative modern-specific insertions might be polymorphic in archaic populations as well; unfortunately, we would need many more available ancient genomes to properly test this. However, in order to estimate the number of potential polymorphic AMH-specific insertions, we took advantage of the large amount of population data provided by the 1000 Genomes project, particularly AFR populations, who are less likely to host Neanderthal-derived genomic traits. Indeed, by randomly sampling AFR individuals we observed that a few samples would be sufficient to identify the vast majority of the archaic-specific polymorphic insertions, reaching a plateau at *n* = 20. Similarly, ~ 45% of the putative species-specific insertions were shown to be polymorphic (Additional file 9C). On the other hand, we observed that the 321 detected RIs that were present in AMH and absent in both archaic genomes fall below the ~ 45% threshold identified with the above procedure (considering polymorphisms against both HD and HN individually). This fact, together with the observation that HD and HN genomes are more divergent than two randomly-chosen AMH genomes [38], suggests that the above mentioned 321 insertions may be considered as reliable and truly AMH-specific.

### AMH-specific RIs in present-day populations

The fact that the detected 321 AMH-specific RI are present in the human reference sequence (GRCh37-hg19) does not necessarily imply that they are fixed in all human populations. We therefore evaluated their distribution in present-day populations by comparing the coverage of the unique 3′ and 5′ flanking regions with that of the RI/flanking interface in 1000 Genomes Phase 3 data (Additional files 9D and 12; more details in METHODS). This analysis revealed that, of the 321 AMH-specific insertions, 24 (7.5%) appear to be present at very high frequency in all modern populations (allele frequency > 85%), while 8 (2.5%) are polymorphic in AFR individuals but extremely common in all non-African populations (allele frequency < 65% in AFR and > 85% in non-Africans), suggesting that their fixation may be related to the Out-of-Africa event.

Interestingly, the patterns of RI distribution reflect known pre-historic and historic migrations and population dynamics of AMH (Additional file 9E). In particular, populations of African descent are the more divergent ones, while the Out-of-Africa ones cluster according to clear phylogenetic/phylogeographic relationships, with the expected exceptions of Puerto Ricans (PUR) and Colombians (CLM) who cluster with European (EUR) populations and not with American (AMR) ones, likely because of admixture during the re-colonization of North and South America [39].

Times to the Most Recent Common Ancestor (TMRCA) were also calculated for 10kbp sequences [40] surrounding each insertion site (Additional file 12, details in METHODS). Of the 24 insertions that are particularly widespread (frequency > 85%) in all modern populations, we selected those showing a TMRCA compatible with the split between AMH and HN/HD (TMRCA < 800 Kya) as potential candidates for selection/spread along the AMH lineage. Accordingly, we identified two RIs (8%), i.e. an AluYg6 insertion in chr1q25.3 that occurred in the gene EDEM3 and an AluYb9 insertion in chr10q25.3 that also occurred within the sequence of the gene SHTN1. Similarly, only one of the 8 AMH-specific insertions that are likely to have increased their frequency post Out-of-Africa, an AluYa5 insertion in chr16q22.1, also displays a recent TMRCA. However, it is worth noting that TMRCA estimates were obtained from all the AFR individuals and not only from the carriers of an insertion; therefore, they must only be considered as a general indicator of the “age” of a given site surrounding an insertion or, in other words, as an upper-limit for the retrotransposition event itself.

Three Population Composite Likelihood Ratio (3P-CLR) statistic [41] was calculated on the Estonian Biocentre Human Genome Diversity Panel (EGDP) dataset [42], then the regions showing signs of selection were overlapped with 200kbp loci surrounding each insertion. Overlapped regions were then subdivided in percentiles of significance in relation to selective pressure signals. This analysis revealed that 28 (9.2%) out of the 306 AMH-specific RIs autosomal insertional loci are within the top 0.1% of loci subjected to post Out-of-Africa selection (Additional file 13, details in METHODS).

### Genomic features of loci containing AMH- and chimp-specific RIs

The huge amount of genetic and genomic data presently available on modern humans allowed us to perform different exploratory analyses on the AMH-specific RIs and their surrounding genomic loci, aimed at evaluating the possible impact of RIs in our species evolution.

**Fig. 1:**
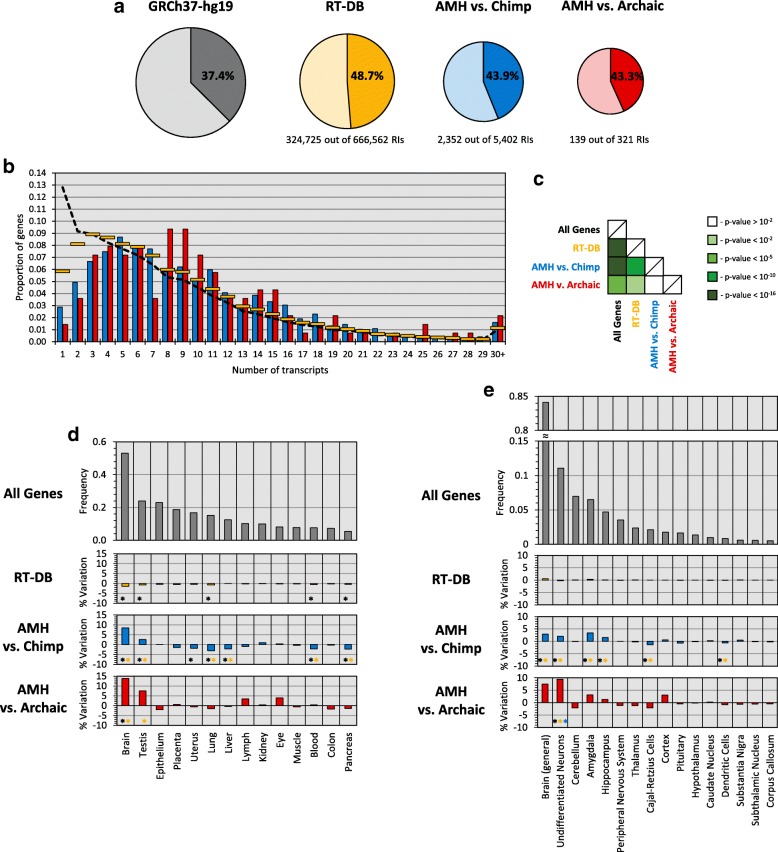
AMH-specific RIs, genes and preferential expression. **a** Proportion of ENSEMBL-annotated genes in the whole reference genome GRCh37-hg19 (grey), proportion of insertions that occurred in annotated genes for RT-DB insertions (yellow), AMH-specific RI vs chimp (blue) and AMH-specific RI vs both HN and HD (red). In each diagram, the darker color denotes the percentage of RIs inserted in genes vs RIs inserted in non-genic regions (lighter color). **b** Proportion of genes per number of annotated transcripts for all ENSEMBL-annotated genes in the reference genome GRCh37-hg19 (black dotted line), for genes with RT-DB insertions (yellow lines), for genes containing AMH-specific RIs vs chimp (blue bars) and for genes with AMH-specific RIs vs both HN and HD (red bars). **c** Table showing statistical significance of the differences between the series of Fig. 3B, calculated with Kolmogorov-Smirnov tests; white is for non-significant *p*-value, light-green is for p-value < 10^− 2^, pea-green is for p-value < 10^− 5^, emerald-green is for p-value < 10^− 10^, dark-green is for *p*-values < 10^− 16^. **d** Proportion of all human genes showing preferential expression in different tissues (grey bars); % increase or decrease in absolute proportions for preferential tissue expression of genes with RT-DB insertions (yellow bars), genes containing AMH-specific RIs vs chimp (blue bars) and genes with AMH-specific RIs vs both HN and HD (red bars). Black asterisks mark significant differences between the series and all human genes while yellow asterisks mark significant differences between the series and genes that contain RT-DB insertions. **e** Proportion of all human genes showing preferential expression in the brain divided by neural regions (grey bars); % increase or decrease in absolute proportions for preferential neural expression of genes with RT-DB insertions (yellow bars), genes containing AMH-specific RIs vs chimp (blue bars) and genes with AMH-specific RIs vs both HN and HD (red bars). Black asterisks mark significant differences between the series and all human genes, yellow asterisks mark significant differences between the series and genes containing RT-DB insertions, blue asterisks mark significant differences between the series and genes containing AMH-specific RIs vs chimp

First, we compared selected datasets of RIs (AMH-specific vs chimp and AMH-specific vs both archaic species) with the ENSEMBL gene annotation [43] of the reference sequence GRCh37-hg19, using the reference insertions of the aforementioned RT-DB database as a control. We determined that 15,367 genes contain insertions of RT-DB (48.7% of the insertions), 1779 genes contain intronic AMH-specific vs chimp RIs (43.9% of the insertions) and AMH-specific RIs occurred in 139 introns of genes after the split with HN/HD (43.3% of the insertions) (Fig. 1a). In general, these data suggests that RT-DB insertions occurred in genes and gene-related sequences, or have been maintained throughout evolution in those sequences, ~ 30% more frequently than randomly expected in respect to gene-size/genome-size (*p*-value < 10^− 16^). As for AMH-specific RIs, their behavior seems coherent (if slightly lower) with the whole RT-DB dataset, in fact they occurred in genes ~ 17% more frequently than expected both vs chimp and vs HN/HD (*p*-values < 10^− 16^ and < 0.05 respectively), testifying the good performance of both our RIs identification method and validation procedure.

We also retrieved ENSEMBL annotation of genes in the chimpanzee reference sequence PanTro5 and generated, similarly to RT-DB for AMH, a database of recent RIs in chimps (defined as RT-DB Chimp). We then compared the proportions of chimp-specific RIs that occurred within genes to those of RT-DB Chimp and of all genes in the chimpanzee genome. Results show that 35% of the chimpanzee genome is annotated as gene-related, while 41.2% of RT-DB Chimp RIs and 39.4% of chimp-specific RIs occurred within genes (Additional file 14A). Both RIs lists occurred within gene sequences more than randomly expected (both *p*-values < 10^− 16^), but they also are present within genes significantly less than their AMH counterparts (p-values < 10^− 5^ in respect to both RT-DB and AMH-specific RIs vs. Chimp).

In addition, the ENSEMBL gene-annotation data revealed that, in general, the majority of genes in AMH genomes tend to have a low number of annotated transcript/splicing variants, with a decreasing trend between the proportion of genes and the number of transcripts (7.584 on average; mode: 1 transcript/gene). Intriguingly, the comparison of genes containing RIs in the human lineage with all others present in AMH genomes (Fig. 1b-c) revealed an average increase in the number of transcript and splicing variants for those genes that contain RT-DB insertions (8.428 on average, mode: 3 transcripts/gene; *p*-value < 10^− 16^). Notably, this trend increases further when analyzing RIs that likely inserted after the split with chimps (9.728 on average, mode: 5 transcripts/gene; *p*-value < 10^− 16^) and after the split with HN/HD (9.863 on average, mode: 8.5 transcripts/gene; *p*-value < 10^− 6^). Consistently, genes containing AMH-specific RIs, both vs Chimp and vs HN/HD, also have more annotated transcripts than genes containing RT-DB insertions (*p*-values < 10^− 11^ and < 0.005 respectively).

The same pattern can be observed for genes containing chimp-specific insertions (3.182 annotated transcripts on average, mode: 2 transcripts/gene; p-value < 10^− 16^) and RT-DB Chimp insertions (2.436 transcripts on average, mode: 1 transcript/gene; p-value < 10^− 16^), in respect to all genes annotated in the PanTro5 reference sequence (1.851 transcripts on average, mode: 1 transcript/gene) (Additional file 14B-C). This trend, however, is much less pronounced in chimps than in the genome of AMH.

We finally performed further analyses on genes containing human RT-DB insertions in order to exclude a possible bias for gene length in the previously reported results. Indeed, a clear correlation is present between number of RIs and number of transcriptional variants (Rho = 0.284, p-value < 10^− 16^), but a strong correlation also exists between gene length and both parameters (Rho = 0.799 with number of RIs, Rho = 0.267 with number of annotated transcripts, both *p*-values < 10^− 16^). Thus, we performed a partial correlation test between the number of RIs and transcript variants of genes in respect to their length, which resulted in a statistically significant association of the first two parameters even after accounting for the third (Rho = 0.122, *p*-value < 10^− 50^). This test was repeated considering only genes containing AMH-specific RIs absent in chimps and AMH-specific RIs also absent in both HN and HD: in both cases the correlation between number of RIs and number of transcripts after accounting for gene length was confirmed (Rho = 0.196 with *p*-value < 10^− 16^ and Rho = 0.240 with *p*-value < 0.005 respectively).

### Preferential expression of genes containing AMH-specific RIs

We then retrieved functional annotation data from DAVID Bioinformatics Resources v6.8 [44] and we obtained tissue-specific preferential gene expression information for 15,126 out of 15,367 genes with insertions from RT-DB, 1721 out of 1779 genes in which retrotransposition events occurred in the human lineage after the split with chimps and 124 out of 139 containing insertions absent in both HN and HD. Comparisons among these data show that genes containing human-specific RIs tend to be more expressed than others in specific tissues (Fig. 1d). In general, genes containing RT-DB insertions tend to follow the same expression profile of all human genes, albeit with a slight under-expression in some tissues (*p*-values < 0.05). However, genes in which AMH-specific RIs occurred after the split with chimpanzees are more expressed than average in the brain and testis (+ 8.5% and + 2.7% in absolute proportions respectively; p-values < 10^− 12^ and < 10^− 2^), while being less expressed in the uterus, lungs, liver, blood and pancreas (decreases between − 1.8% and − 3.1% in absolute proportions; all *p*-values < 10^− 2^). Finally, genes with AMH-specific RIs absent in both HN and HD are significantly more expressed in the brain and, with respect to genes containing RT-DB insertions, in testis (+ 13.8% and + 7.5% in absolute proportions, *p*-values < 5 × 10^− 3^ and < 0.05).

We next focused on genes preferentially expressed in the brain (Fig. 1e). Our results show that genes containing RT-DB insertions follow the same expression pattern of all human genes. Instead, genes with AMH-specific vs. chimps RI are generally highly expressed in the brain and seem to be even more expressed than average in the amygdala and hippocampus, as well as in undifferentiated neurons (+ 3.4%, + 1.5% and + 2.0% in absolute proportions respectively, p-values < 10^− 4^ for the amygdala and < 0.05 for both hippocampus and undifferentiated neurons). At the same time, they show less expression in Cajal-Retzius and dendritic cells (− 1.4% and − 0.7% in absolute proportions, p-values < 10^− 3^ and < 0.05). Finally, genes containing AMH-specific RIs absent in both HN and HD are significantly more expressed than average in undifferentiated neurons (+ 9.4% in absolute proportions, p-value < 10^− 2^).

Having previously evidenced a correlation between number of RIs present in genes and their length, we tested the possibility that genes showing preferential expression in specific tissues could exhibit a bias in relation to their length. We thus performed a pairwise Wilcoxon test between all series of lengths of genes preferentially expressed in the different tissues. This test showed a relative homogeneity of length of the various groups of genes, albeit with some pairwise confrontations resulting in statistically significant difference between the pairs of series (Additional file 15A).

Furthermore, we used the obtained matrix of p-values of the pairwise comparisons as a matrix of distances between the series in order to perform a cluster analysis on the different categories (Additional file 15B). This resulted in the distinction of 3 major groups of genes based on their length distribution: genes preferentially expressed in the Eye, Kidney, Epithelium, Brain and Testis are generally longer, genes expressed in the Pancreas, Blood, Lung and Muscle are shorter, while genes expressed in the Colon, Lymph, Liver, Placenta and Uterus fall in between.

Focusing on genes preferentially expressed in the Brain, which have been highlighted as containing more AMH-specific insertions, they do not seem significantly larger than other genes and instead form a cohesive group with genes expressed in other tissues that do not seem to contain as many AMH-specific RIs. Thus, these results seem to imply that the impact of gene length on previous analyses (if any) was negligible.

### Gene ontology of genes containing AMH-specific RI

**Fig. 2:**
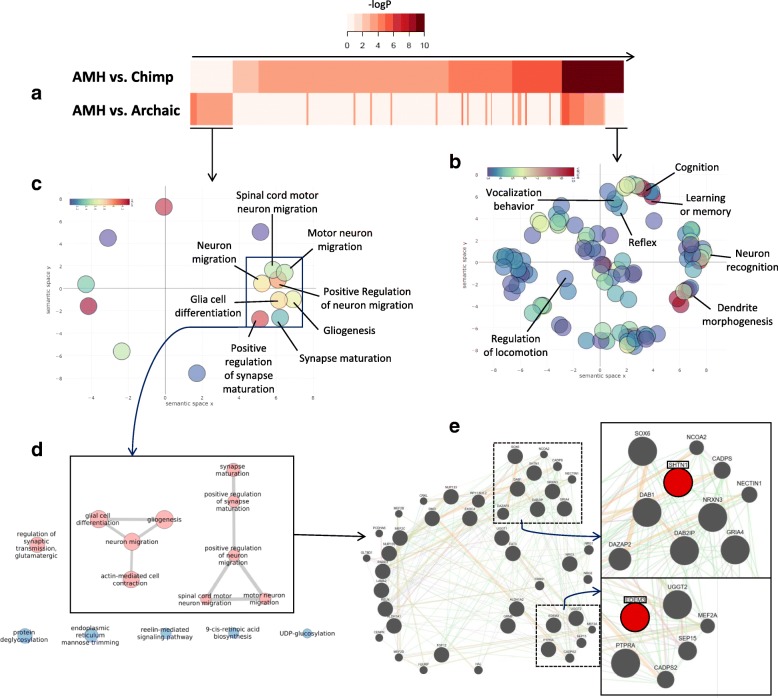
GO analyses of genes containing AMH-specific RIs. **a** Heat maps representing -log (p-values) of GO terms associated with genes that contain AMH-specific RIs vs chimp (top) and AMH-specific RIs vs both HN/HD (bottom), ordered for increased significance in the top row. **b** Scatterplot representation of the identified Hominina-specific GO terms. The x and y coordinates of the circles were derived from the Revigo analysis, based on multidimensional scaling on the matrix with the GO semantic similarity values. The functional categories associated with genes that form networks are highlighted and labeled. **c** Scatterplot representation of the identified sapiens-specific GO terms. The x and y coordinates of the circles were derived from the Revigo analysis, based on multidimensional scaling on the matrix with the GO semantic similarity values. The functional categories associated with genes that form networks are highlighted and labeled. **d** Functionalities of sapiens-specific GO terms associated in networks (if applicable). Red terms are neural-related while blue terms are not. **e** Gene network of genes containing AMH-specific RIs absent in both HN and HD with neural functionalities. The larger the circle, the more the gene represented by it has interactions with other genes in the network. The sub-network showing strong interactions with the gene SHTN1 is highlighted in the top-right, while the sub-network with more interactions with the gene EDEM3 is highlighted in the bottom-right

In order to examine the functionality of genes in which insertions occurred, Gene Ontology (GO) analyses were performed on all genes containing AMH-specific RIs, both vs Chimp and vs HN/HD. ToppCluster analyses [45] revealed that, of the 238 GO terms identified between the two lists, 175 GO terms (73.5%) were overrepresented in genes that contain AMH-specific RIs vs chimp, whereas 23 (9.7%) were overrepresented in genes containing AMH-specific RIs vs both HN/HD (Fig. 2a, Additional file 16). Next, we selected the GO terms that were enriched in one group of genes and not in the other as lineage-specific functionalities that might correspond to different moments in the evolution of the human lineage, i.e. Hominina-specific GO terms (for terms enriched only in genes with AMH-specific RI vs chimp) and sapiens-specific GO terms (for terms enriched only in genes with AMH-specific RI vs both HN/HD). Interestingly, semantic similarity of Hominina-specific GO terms showed that the most enriched functionalities of genes containing AMH-specific RI vs chimp are related to cognition, learning and memory capabilities, vocalization behavior, neuron recognition, dendrite morphogenesis, reflexes and regulation of locomotion (Fig. 2b). Interestingly, these functionalities associate in networks involving a large number of genes containing AMH-specific RIs vs chimp. Furthermore, for enriched sapiens-specific GO terms, all functionalities associated in networks are neural-related: synapse maturation and its regulation, neuron maturation and migration, gliogenesis and glia differentiation (Figs. 2c-d). Genes associated with these GO terms also form a complex network of interactions (Fig. 2e). It is worth noting that two of the genes with the larger amount of interactions in this network are SHTN1 and EDEM3 (1st and 11th scores in order of significance), which contain the previously identified (see above) AluYg6 RI (chr1q25.3) and the AluYb9 RI (chr10q25.3) respectively.

### Evidences of RI contribution in the molecular differentiation of AMH

**Fig. 3:**
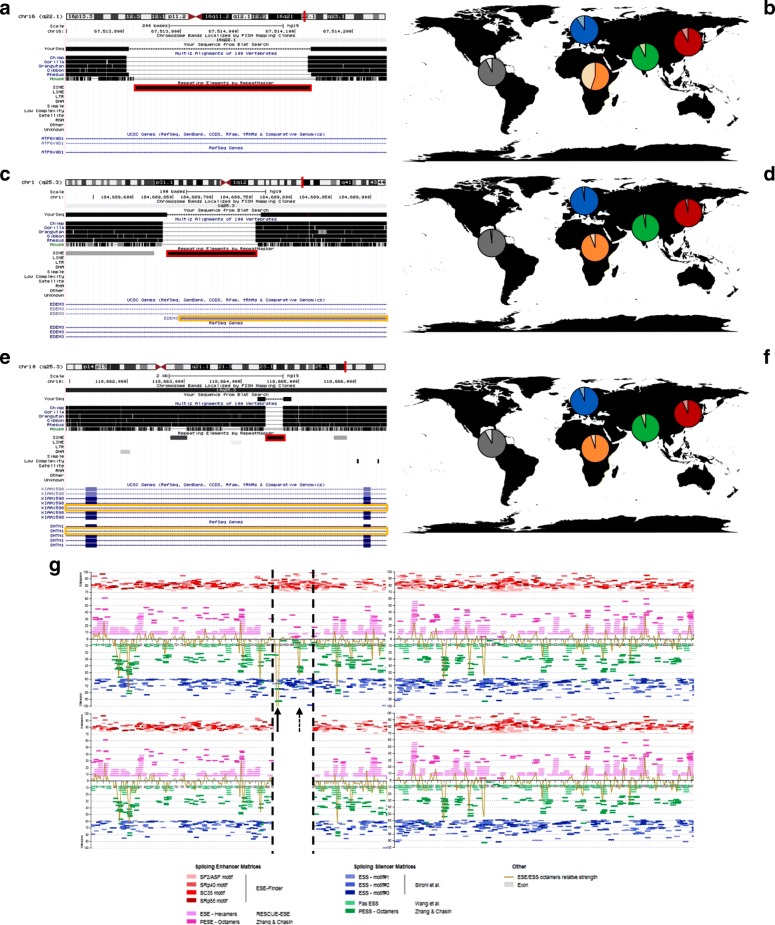
Impact of AMH-specific RIs. **a**-**b** Annotation of the genomic location and distribution in present-day populations of the AluYa5 insertion on chr16q22.1. The insertion is highlighted in red in **a**; in **b**, for each diagram, a darker color indicates the presence of the RI and a lighter one its absence. **c**-**d** Annotation of the genomic location and distribution in present-day populations of the AluYg6 insertion on chr1q25.3. In **c**, the insertion is highlighted in red and a yellow rectangle highlights an alternative transcript that terminates precisely at the poly-A tail of the RI; in **d**, for each diagram, a darker color indicates the presence of the RI and a lighter one its absence. **e**-**f** Annotation of the genomic location and distribution in present-day populations of the AluYb9 insertion on chr10q25.3. In **e**, the insertion is highlighted in red and yellow rectangles highlight annotated alternatively-spliced products for the gene in which the insertion occurred; in **f**, for each diagram, a darker color indicates the presence of the RI and a lighter one its absence. **g** Splicing prediction in the sequence corresponding to filed allele (top, containing the intron and the AluYb9 insertion on chr10q25.3) and in the sequence corresponding to the empty allele (bottom, containing just the intron). The sequence is oriented in the same transcriptional sense orientation of the gene, black dotted lines highlight the position of the RI. Pink and red lines represent Splicing Enhancer Matrices, green and blue ones Splicing Silencing Matrices; ochre lines represent the combined strength of Enhancer/Silencing Matrices on the sequence. Arrows highlight silencing signal peaks that occur precisely in the RI sequence

Since AMH-specific RIs might increase the variability of transcripts and tissue-preferential expression of the genes in which they inserted, we next characterized in greater detail the insertional loci of the three “recent” insertions with a peculiar population distribution that were identified above (see “AMH-specific RIs in present-day populations”). The first one, an AluYa5 RI in chr16q22.1 (Fig. 3a-b), is polymorphic in AFR populations (average frequency of 55%), but fixed or almost fixed in all non-African populations (with the highest difference in frequency between African and non-African populations). Although no role in functional alteration was detected for this RI in its insertional locus, the insertion is associated with signs of post Out-of-Africa selection, as revealed by the 3P-CLR selection estimate that places its genomic locus in the top 0.1% loci. The second RI analyzed, an AluYg6 insertion in chr1q25.3, inserted in gene EDEM3 (mentioned above) and is estimated to be fixed or almost fixed in all AMH populations, while completely absent in chimps, HN and HD, as well as in other primates (Fig. 3c-d). Transcript annotations for this gene show a shorter EDEM3 alternative transcript ending precisely in correspondence with the poly-A tail of the AluYg6 insertion, resulting in exonization of this RI. This alternative EDEM3 transcript, which is not annotated in chimpanzees, is probably a direct consequence of the AluYg6 insertion. The third and last RI analyzed is an AluYb9 RI in chr10q25.3 that inserted in the 15th intron of the gene SHTN1, antisense in respect to the gene’s transcriptional directionality (Fig. 3e-f). This RI has the highest level of interaction in the previously identified network of neural genes and is widespread in all AMH populations and absent in HN, HD and chimp genomes, as well as in other primates.

The gene SHTN1 has various annotated transcriptional/splicing variants, two of which lack the first two exons flanking the intron where the Alu insertion occurred. Intriguingly, the analyses of this intron with Human Splicing Finder 3.0 [46], both as an empty allele and as a filled allele with the AMH-specific AluYb9 insertion, revealed that differences in the predicted Splicing Enhancing/Silencing Matrices are present between the two sequences, suggesting the presence of putative splicing-silencing peaks in the filled allele. The strongest peak is located precisely in the inserted AluYb9 sequence (Fig. 3g), suggesting that the Alu insertion may induce a splicing-silencing effect on the SHTN1 gene.

## Discussion

In AMH, retrotransposition has been studied mostly for its mutagenic effects and implications in disease insurgence. On the other hand, knowledge about the molecular evolution of our genome relies mostly on markers such as SNPs, short InDels and large Copy Number Variants (CNVs), while the role of repetitive/complex regions of the genome is poorly understood. Among others, the complexity of analyses involving repetitive sequences and structural variations in conjunction with the widely used NGS sequencing technology is a challenging task. However, evidences from various Eukaryote organisms suggest that retrotransposition might play an important role in speciation and molecular evolution of genomes [1, 47].

In this study, we evaluated the possible impact of RIs on the differentiation processes that occurred in the human lineage and especially during AMH evolution. In order to do this, we first identified putative species-specific RIs across the genomes of AMH, HN, HD and chimpanzees (Table 1). Importantly, all the identified insertions are inheritable and have actually been transmitted throughout generations, meaning that they are not somatic insertions occurred in specific tissues, but rather germline/pre-germline RI events.

The identified species-specific RIs reveal a rate of accumulation in the human lineage (0.6–0.8 insertions/Ky) which is ~ 2.5× higher than that of chimpanzees (0.29 insertions/Ky). As previously observed [32], these data suggest that n-LTRrs might have impacted our genome throughout the evolution of the human lineage more than they have affected the chimp lineage, despite chimpanzees’ shorter generation time. However, the relatively lower content in RIs of chimps may, at least in part, be ascribed to differences in the assembly of their reference sequence. Finally, we note that the impact of RIs described in this study is just a minor repertoire of the putative effect that RIs can exert on genome structure and regulation, as i) our study is limited to the identification of canonical RIs on limited sequencing information from Neanderthal, Denisovan and Chimpanzee genomes; and ii) in this study we only analyzed the impact of RIs in *cis*, although RIs are known to impact gene expression and genomic architecture both in *cis* and in *trans* [48].

### AMH-specific RIs and functional variability increase at insertional loci

Focusing on the insertions that are specific to AMH, one of our main results is the strong association highlighted between RIs that integrated in genes and the increase in the number of annotated transcript for the genes in which the insertion occurred (Fig. 1b-c). While this is true for all RT-DB insertions and the corresponding genes, the effect seems even higher for RIs that are exclusive to AMH. We may interpret this as a sign of target-preferentiality of retrotransposons in general and particularly in the human lineage. RIs might thus preferentially target genes with a high variety of transcripts. Another possible explanation is that, just by random chance, longer genes might accumulate more RIs than shorter ones, at the same time exhibiting a higher variety of transcripts. While there definitely is a strong correlation between gene length and both number of insertions and number of annotated transcripts, this does not seem to explain all of the observed variability in human genes annotation. Indeed, a correlation between number of identified RIs and variety of transcripts remains even after accounting for gene length. This observation, together with the characteristics of N-LTRr sequences and their possible effects upon integration [49, 50], strongly suggest that at least some part of the increase in the variety of transcripts is an effect, and not a cause, of the accumulation of new retrotransposition events. Therefore, we could speculate that the new transcript/splicing variants of genes containing AMH-specific RIs has led to an increase in their functional complexity.

The aforementioned trend is also visible in the genome of chimpanzees, albeit in a much less pronounced fashion (Additional file 14B-C). The difference between genes containing AMH- and Chimp-specific RIs might be, at least in part, attributable to biological reasons, although the difference in transcriptional variant annotation in the two genomes could bias statistical comparisons between them (~ 58,300 transcript variants annotated in PanTro5 vs > 200,000 in GRCh37-hg19) [43].

We then observed that genes containing AMH-specific RIs tend to be preferentially expressed in specific tissues (Fig. 1d-e). In the human lineage, these genes are especially likely to be expressed in the brain, with an enrichment of > 26% compared to its preferentiality for all human genes. In particular, genes with AMH-specific insertions vs chimp are more expressed in the amygdala, hippocampus and undifferentiated neurons (up to > 52% in respect to each cell-type/tissue expectation), while after the split with HD and HN the enrichment of preferential expression occurs specifically in undifferentiated neurons (> 85% in relation to their general baseline). These results seem to be unaffected by the length of the genes expressed in the different tissues. In fact, genes expressed in the human brain are not significantly longer than those expressed in other tissues and, instead, when grouped by their dimensions, form a coherent cluster with genes preferentially expressed in the eye, kidney, epithelium and testis.

These results indicate a strong association between RIs and neural genes in the lineage of AMH. Indeed, GO analyses revealed a consistent pattern of neural-related functionalities for genes containing RIs, which is consistent with the aforementioned tissue-specific preferential expression. Interestingly, Hominina-specific GO terms of genes containing AMH-specific RIs vs chimp are highly related to biological and ethological processes that occurred during the differentiation of hominids after the split from chimpanzees, including: neuronal signaling, cognitive capacity, vocalization behavior, reflexes and locomotion regulation (Fig. 2b). Furthermore, the most relevant functionalities associated with sapiens-specific GO terms all relate to glia differentiation, synapse maturation and neuron maturation and migration (Fig. 2c-d). These functionalities, associated with the preferential expression in undifferentiated neurons of genes that carry AMH-specific RIs absent in both HN and HD, might reflect the importance of these genes in human neural differentiation processes, especially due to the fact that the identified RIs are not somatic nor specific to those tissues, but instead inheritable.

We could therefore speculate that the aforementioned increase in transcript variability of specific genes, seemingly induced by RIs, may be tied to the increase in functional complexity of the human brain that occurred all throughout our evolutionary lineage [51].

### Population distribution of RIs and natural selection

These possible variability-increasing effects of RIs in our lineage and their specific relevance in neural genes, theoretically, should have been subjected to natural selection. Among the identified 321 AMH-specific RIs, the most likely candidates for an adaptive effect on their carriers are those insertions showing widespread diffusion across all AMH populations or whose distribution reflects a strong phylogenetic/phylogeographic pattern (e.g. fixation post Out-of-Africa). We therefore compared the identified RIs in this study with the genomic variability of present-day human populations provided by the 1000 Genomes Consortium data. AMH-specific RIs, according to TMRCAs, seem to have occurred between > 6.5 Mya (i.e. before the split with the chimpanzee lineage) and present day (Additional file 12). However, these time estimates must only be intended as upper limits for the actual times of the insertions occurrence. Indeed, a large portion of identified putative HN- and HD-specific insertions was shown to be polymorphic to various degrees in present-day populations. In particular, 8 archaic-specific insertions are absent in all African individuals and present only in non-African populations. Thus, we speculate that these RIs might have introgressed in AMH via interbreeding with the archaic species after *Homo sapiens* migrated out of Africa. Conversely, given the documented presence of Neanderthal introgressed sequences within the genome of Eurasians [27, 28, 32], which in turn forms the majority of the human reference sequence, some HN- and HD-specific insertions might be present on the human reference due to archaic introgression that escaped our detection.

As expected, RIs seem to have been selectively neutral and polymorphic throughout AMH populations, although in some instances they show traces of selection only a long time after their putative occurrence (Additional files 12 and 13). Amongst all RIs that are present at high frequencies in modern populations, a few of them show a peculiar geographic distribution characterized by polymorphism in Africa and almost fixation in non-African individuals. We hypothesize that these RIs could have reached high frequencies through genetic drift or selection following the Out-of-Africa event. In both cases, these insertions predate the spread of AMH out of Africa. In particular, the AluYa5 insertion in chr16q22.1 identified in this study (Fig. 3a-b) has a TMRCA of ~ 300 Kya and displays rapid frequency increase in non-African populations, with its surrounding locus being in the top 0.1% 3P-CLR loci. Therefore, we speculate that this insertion (and/or its surrounding locus) was actually subjected to selection post Out-of-Africa, possibly hundreds of Ky after the insertion itself occurred. However, due to the lack of specific methodologies of time estimation and selection for RIs, our data is only an approximation for both the putative age of an insertion and selective pressures acting on an insertional locus.

In sum, we hypothesize that RIs might occur in a genome and be maintained randomly within a population under neutral selective pressures. At later times, because of population dynamics or environmental changes, an insertion and the putative novel functional variants it generated might be co-opted, even only in specific tissues/cell-types, and undergo non-neutral selective pressure in a similar manner as previously reported cases of “soft” selective sweeps detected with SNPs analyses [52, 53]. On an evolutionary timescale, this process seems more likely than insertions having a strong functional-alteration effect immediately upon integration. In fact, most functional regions of a genome are highly conserved and functionality-altering effects would likely be disease-inducing and selected against alleles carrying the RIs. Additionally, genetic drift might also play an important role in the maintenance/diffusion of RIs in human populations, particularly concerning Out-of-Africa bottlenecks.

This interpretation is also consistent with differences in the percentage of RIs targeting genes observed in RT-DB with respect to AMH-specific RIs, both vs chimp and vs HD/HN (Fig. 1a). Indeed, these three datasets of retrotransposition events are progressively smaller subsets of the same starting pool of insertions and reflect progressively shorter timescales. Importantly, the effects of a retrotransposon insertion can be co-opted even a long time after the insertion itself occurred, creating new functional variants; thus, a dataset of older insertions (on average) is more likely to show annotated functional variants in a modern genome than a dataset of relatively younger insertions. Furthermore, our results regarding Chimp-specific RIs and their occurrence within genes (Additional file 14A), together with the observed different rate of RI accumulation between AMH and chimpanzees, suggest that this process was likely already in place before the AMH-Chimp split and that it increased specifically in the human lineage.

It is also worth noticing that, to the best of our knowledge, the only process that can remove RIs after their occurrence is recombination. Its dynamics and the negative selection that ectopic recombination is subjected to can drive the maintenance of RIs in gene-rich regions and may tend to lower the number of insertions in heterochromatic regions [54, 55]. These processes could partially alter the interpretation of our findings; however, the interaction of the effects of recombination and the potential generation of new molecular variants due to RIs can synergize in increasing genomic variability in evolutionary time-frames. This interpretation seems to be coherent with the previously proposed evolutionary dynamics involving generation of variability, co-option and soft selective sweeps, rather than strong functional alterations followed by rapid hard selective sweeps.

### Impact of RIs in modern humans

Previous studies revealed how retrotransposons can influence the regulation of the loci in which they inserted in a myriad of ways [1]. Besides the activity of the sense and antisense LINE-1 promoters contained within full-length LINE-1 s [7, 56, 57] and the epigenetic silencing of retrotransposon sequences mediated by DNA methylation [58] or histone modifications [59, 60] that can directly impact gene expression, other common effects of RIs on genes in which they inserted include premature transcript termination [8, 61] and alternative post-transcriptional processing of genes [49, 50, 62, 63]. Some of these functional impacts are generated by RIs inserted in genes because of the A/T richness of the LINE-1 sequence [8] and due to the presence of a poly-A tail at the 3’end of the retrotransposon insertion, which can increase the repertoire of transcripts produced from the gene containing the insertion (i.e., generating alternative transcripts). Similarly, Alu elements carry a functional polymerase-III promoter that can directly influence gene expression [64]; furthermore, some Alu insertions can affect the expression of genes in which they inserted by additional mechanisms [65–68]. Indeed, the AluYg6 insertion on chr1q25.3 identified in this study (Fig. 3c-d) seems to directly affect EDEM3 gene expression, as an alternative annotated transcriptional variant in humans terminates precisely in the AluYg6 poly-A tail. Intriguingly, EDEM3 belongs to a group of proteins that accelerate degradation of misfolded or unassembled glycoproteins in the Endoplasmic Reticulum [69]. The EDEM3 gene also has a large number of associations in the functional network of neural-related genes containing AMH-specific insertions that are absent in both HN and HD, suggesting a strong relevance for EDEM3 in this network (Fig. 2e).

Another important effect that RIs can exert on gene expression, especially antisense insertions in respect to the gene’s transcriptional orientation, is the alteration of the post-transcriptional processing of mRNAs, which can result in alternatively-spliced RNAs [70]. Indeed, the identified AluYb9 insertion on chr10q25.3 that occurred in the 15th intron of the SHTN1 gene (Fig. 3e-f-g), is likely influencing the post-transcriptional processing of SHTN1 mRNAs. Although based on computational predictions, the splicing-silencing peaks associated with the allele containing the AluYb9 sequence suggests that this insertion may affect the post-transcriptional processing of this gene. Therefore, we hypothesize that the AluYb9 sequence might induce alternative splicing of SHTN1 transcripts. In sum, these data illustrate how intronic RIs can contribute to the generation of novel functional variants exclusive to AMHs. Additionally, the gene SHTN1 is highly expressed in the human brain and is involved in the generation of internal asymmetric signals required for neuronal polarization and neurite outgrowth [71]; it is, as well, the gene with most interactions and relevance in the detected network of neural-related genes containing AMH-specific RIs vs both HN and HD (Fig. 2e). In addition, previous studies based on SNPs identified SHTN1 as a target for positive selection in AMHs after the split with HN and HD [31]. Accordingly, the genomic locus of the AluYb9 insertion displays a TMRCA of ~ 560 Kya, which is consistent with possible positive selection and spread after the split between the AMH and HD/HN lineages. Thus, the above-mentioned novel functional variants might have contributed to the establishment of the selective process on this neural gene, which in turn may have affected our species differentiation.

## Conclusions

The results presented in this study suggest that non-LTR retrotransposons-mediated processes might have played a significant role in recent human evolution. RIs presence/absence polymorphisms in present-day populations can be useful phylogenetic markers and highlight interactions and population dynamics that occurred after the separation from the chimpanzee lineage. RIs display patterns of maintenance and diffusion in modern populations that reflect continuous generation of genomic variability. As the new variants can be co-opted at a later moment, selective pressures can arise possibly inducing frequency increase or purification of those variants. Indeed, non-LTR retrotransposons activity results in an enrichment of pre- and post-transcriptional variants of genes in hominids and can directly generate new functionalities for human genes. This process is particularly evident in the pool of most-recent RIs (AMH-specific ones). In fact, these new variants were probably co-opted throughout the evolution of AMH and genes producing those variants are preferentially expressed in specific tissues. Co-option of putatively RI-induced variants seems to have occurred especially in the brain, where they are related to neuronal maturation and migration, as well as synaptic-recognition; they are also associated to functionalities such as cognitive capability, vocalization behavior and locomotion regulation. Thus, RIs are possibly involved in the differentiation processes of the human brain and its increase in complexity that took place all throughout the evolution of the human lineage. In some instances, as for the AluYg6 insertion on chr1q25.3 and the AluYb9 insertion on chr10q25.3, the effects of these RIs on their target in *cis* might have been key contributors to the molecular differentiation of AMH genomes.

Indeed, since our study is limited to a few Neanderthal, Denisovan and Chimpanzee genomes, and because only RI-impacts in *cis* could be analyzed, the impact of non-LTR retrotransposonsons on human evolution reported in this study probably reflects only the tip of a much larger iceberg. However, the contribution of non-LTR retrotransposition, whose understanding still needs to be further developed, is starting to shed light on the variety and complexity of RI-driven evolutionary processes that shaped our genome and will continue to influence our evolution in the future.

## Methods

### RI identification between AMH and HN/HD

The methodology is schematically described in Additional file 1. Letters in the text correspond to conceptual steps in the scheme. It uses established bioinformatic tools such as the BLAST+ package [72], ABySS [73], BEDTools [74] and RepeatMasker [75], implementing them with custom R or Perl scripts for filtering, conversion and general data management. The methodology was designed on a simulated dataset composed of 100 random locations in the human reference genome GRCh37-hg19, both genic and intergenic. In 50 of the 100 random loci, an RI was artificially added simulating a non-reference retrotransposition event, while in the other 50 an existing reference RI was artificially removed reconstructing an empty (pre-insertional) site. RI artificially added or removed accounted for Alu, LINE-1 and SVA elements, both full length and truncated. All the thresholds used during the procedure (most notably blastn ones) were defined in order to retrieve all simulated insertions. After successfully completing the simulated procedure, the same methodology and thresholds were optimized and finally applied to real genomic data. All ancient DNA sequencing reads were considered as single-ended for the purpose of RI/flanking sequences interface identification.

Step 1. We retrieved consensus sequences of the most recent non-LTR retrotransposons from RepBase [36] (AluYa5, AluYb8, AluYb8a1, AluYb9, AluYb10, AluYb11, AluYk13, LINE-1HS, SVA_A, SVA_B, SVA_C, SVA_D, SVA_E, SVA_F), as well as the genomic material of the species to compare (reference sequence GRCh37-hg19 for AMH, the raw reads of the DNA sequencing for both HN and HD). Specifically, the genomes analyzed in this study are those of two individuals, a Neanderthal and a Denisovan, who lived in the Denisova cave at different times [27, 29]. These two genomes were selected for their relatively high coverage and ready availability. The selected retrotransposon sequences were identified in both genomes using blastn (A,B), setting the identity parameter to 95%. This was done in order to allow the identification of retrotransposons diverging as much as 5% from their consensus sequence. Because of the repetitive nature of TEs, each insertion has been associated to its unique genomic target. For AMH, this was done by extending TEs matches by 100 bp in the 3′ direction and in the 5′ direction in the reference sequence (3′ and 5′ flanking sequences). The same could not be done for the archaic DNA, having reads averaging 100 bp as a starting point. Many new retrotransposon insertions are 5′ truncated, thus the length and 5′ end of an insertion is not known beforehand. For this reasons, we implemented a custom R script to select the reads that matched at least 30 bp of the 3′ end of the retrotransposon’s sequence and that had at least 30 bp of flanking sequence in the 3′ direction. In order to take account of differential length of the insertions poly-A tails in respect to the consensus sequences we allowed for 25 bp of margin between insertions and flankings. The sequences of the 3′ ends of insertions with their respective flankings were then compared between the two species using blastn, with identity parameter set to 95% (C). Sequences that were present in one species’ DNA and not in the other were selected as putatively species-specific insertions, thus producing two lists: putative archaic-specific insertions 3′ portions (D) and putative modern-specific insertions 3′ portions (E).
Step 2.1. The 3′ flanking portions of the putative-archaic specific insertions were used to identify their respective “empty” (pre-insertional) sites in the AMH genome, aligning them with blastn (identity 95%). The selected 3′ portions of the empty sites were extended in the 5′ direction, thus retrieving the sequence corresponding to the 5′ flankings to the putative archaic-specific insertions. The whole empty sites from the AMH genome (200 bp long) and their 3′ and 5′ portions (both 100 bp) were classified in separate sets, using shared codes for sequences belonging to the same site (F). Then, the 5′ portions of the modern-specific empty sites were identified in the archaic DNA-sequencing reads library, using blastn (identity 95%). We then filtered archaic reads containing a match of at least 30 bp to a modern-specific “empty” site’s 5′ portion and at least 30 bp of non-matching bases 3′ of them. These reads should thus contain both the 5′ flanking site and the 5′ terminal portion of the RI (G). The reads pertaining to the two sets of putative archaic-specific insertions 3′ and 5′ portions were associated to their corresponding modern-specific “empty” sites. This allowed to perform de novo assemblies site-by-site using the software ABySS (parameter k set to 40, H,I). Only sequences that were unambiguously assembled for both the 3′ and 5′ portions and that had a clear match for a modern-specific empty site were kept to produce the final sets of confirmed archaic-specific insertions 3′ and 5′ portions, as well as confirmed empty sites from the AMH reference genome (J).
Step 2.2. The putative modern-specific insertions 3′ portions were extended to cover the full insertion as well as 100 bp of flankings in both directions (K). Archaic DNA reads were matched against the 3′ and 5′ flanking sequences using blastn (identity 95%). Reads with at least 30 bp match to both flanking sequences were selected. By doing this, the selected reads spanned the whole empty (pre-insertional) site (L). After associating the archaic reads corresponding to the putative empty sites to their respective modern-specific insertions, de novo assembly site-by-site was performed with ABySS (parameter k set to 40, M). Only putative modern-specific insertions whose flankings corresponded to an unambiguously assembled archaic empty site were selected as confirmed AMH-specific insertions (N).

After RI identification, all insertions were validated computationally. Putatively modern-specific RI were selected for having only one matching empty (pre-insertional) site unambiguously assembled from ancient DNA reads. All validated AMH-specific insertions and their absence from the assembled archaic empty sites were verified using RepeatMasker. Putatively archaic-specific insertions were instead selected for having unambiguously assembled both portions of each insertions and matching only one empty (pre-insertional) site in the modern reference genome. All archaic-specific insertions 3′ and 5′ portions were verified with RepeatMasker, as was their absence from the modern-specific empty sites. All archaic- and AMH-specific insertions were also verified for presence of the poly-A tail of the inserted element and TSDs flanking the RI (Additional file 1).

### RI identification between AMH and chimpanzee reference sequences and RT-DB

First, we retrieved all RI from element families which are known to have been recently active (all AluJ, AluS and AluY subfamilies, all LINE-1HS and LINE1-PA subfamilies, all SVAs) in the two species reference sequences (GRCh37-hg19 and panTro5) from RepBase [36]. The 5′ and 3′ flanking regions (100 bp) for all retrieved insertions were aligned using blastn (identity 95%) to the genome of the other species in order to find the respective putative empty (pre-insertional) sites. Two matching sequences (at least 85 bp), in close proximity to each other (less than 50 bp), were selected as a putative “empty” site for each “filled” site. These putative empty sites were then aligned back to the first species DNA using blastn (identity 95%) in order to confirm them as pre-insertional loci. After this procedure, we obtained the insertions specific to the first species (i.e. absent in the second) and vice versa.

RT-DB insertions were retrieved from the human reference sequence GRCh37-hg19 and represent all reference insertions of AluS and AluY subfamilies, LINE-1HS, LINE-1PA2, LINE-1PA3, LINE-1PA4 and all SVAs annotated in RepBase [36].

RT-DB Chimp insertions were retrieved from the chimpanzee reference sequence PanTro5 and represent all reference insertions of AluS and AluY subfamilies, LINE-1Pt, LINE-1PA2, LINE-1PA3, LINE-1PA4 and all SVAs annotated in RepBase [36].

### Archaic-specific insertions in 1KG populations and inter-specific RI estimation

All archaic specific insertions loci were checked in 1000 Genomes Phase3.vcf files [37] for the identification of non-reference variants present in modern day individuals. Archaic RI frequency was then averaged in modern populations according to the 1000 Genomes project annotations.

In order to estimate the amount of RI insertions that are polymorphic between populations we checked for the presence of Archaic RI in one AFR individual, then incrementally added other AFR individuals to the comparison. The rate by which polymorphic insertions were identified produced a curve that reaches a plateau after 20 individual confrontations. Applying this model to AMH insertions results in 554 and 376 non-polymorphic between species AMH insertions (vs HN and HD respectively).

### Assessment of AMH-specific RI in 1KG samples and frequency-based population tree

AMH-specific insertions present in the human reference GRCh37-hg19 may be polymorphic within the broader human population. However, lack of aligned reads spanning the insertion is, in itself, necessary but not sufficient to diagnose the absence of a given insertion within an examined resequenced genome. Even if present, indeed, given the high similarity to other copies of the same transposable element elsewhere in the genome, a given insertion may display no aligned reads due to multiple-mapping filterings. To assess presence/absence of a given insertion we therefore estimated the average coverage of the 1100 bps up and down-stream (“surroundings”) of a putative insertion site and compared it with the coverage of the first and last 10 bps within the RI itself (“interfaces”). We therefore avoided any inference based on the coverage of the “core” inserted sequence, since this may have been affected by the multiple-mapping issues described above. We, instead, reckoned that the first and last 10 bps at the interface between the RI and the surrounding loci could be considered unique enough for the mapping algorithm to see them as a single mapping hit. Based on the reads available from the 1000Genomes Phase3.bam files [37] we then considered as:

- “diploid present” an insertion displaying a coverage > 0 at both interface regions and where at least one interface region shows a coverage greater than ½ of the average surrounding coverage;
-“haploid present” an insertion displaying a coverage > 0 at both interface regions and where both the interface regions show a coverage smaller than or equal to ½ of the average surrounding coverage;
- “absent” if at least one of the interface regions or the surroundings have zero coverage.

Our assessment approach is conservative with respect to the presence of a given insertion, since it is designed to overestimate absence. We then calculated population frequencies of presence of any given insertion, based on all the individuals available from 1000 Genomes Phase 3 [37].

For each RI we calculated the absolute delta frequency per each pair of populations and we averaged it for all the insertions. The obtained matrix of average differences in presence/absence of human specific insertions was used to build a neighbour joining tree using the Ape R package [76].

### TMRCA estimates of genetic regions surrounding AMH-specific RI

The time to the Most Recent Common Ancestor (TMRCA) of each 10kbp regions encompassing a given insertion was estimated as described elsewhere [40] based on 1000 Genomes sequences of AFR samples to avoid potential backwards biases due to the documented Neanderthal introgression in Eurasians [28]. All AFR individuals, and not only carriers of an insertion, were used for this calculations.

### 3P-CLR selection estimates for regions surrounding an insertion

For the sites surrounding AMH-specific insertions we aimed at identifying those that underwent positive selection after the split between Africa and Eurasia but prior to population differentiations within Eurasia. To do so, we used the Three Population Composite Likelihood Ratio (3P-CLR) statistic [41], to look for regions in the EGDP dataset [42] that show evidence of selection that likely occurred shortly after the expansion out of Africa. The 3P-CLR statistic assumes a 3-population tree model with no post-split migration. To ensure that the individuals used in the 3P-CLR analyses represent the most basal split within living Eurasian populations, we used for our EAS population only Chinese and Japanese individuals from the Mainland East and Southeast Asia macro-population. The EUR individuals used were a random subset of the South and West Europe and East and North Europe populations. The AFR outgroup population consisted of the Yoruban individuals from the EGDP dataset [42]. Following indications [41], 100 SNPs (with at least 20 SNPs between them) were sampled in each window of length 1 cM. Upon completion of the scan, sampled SNPs were grouped into 200 kb bins that were assigned the maximum 3P-CLR score of the sampled SNPs in the window. Windows containing an AMH-specific insertion site and falling within the top 99th percentile of scores from this 3P-CLR test were considered to be under selection along the shared Eurasian branch.

### AMH- and chimp-specific RIs, genes and preferential expression

Gene- and transcript-annotation tracks for the human reference genome GRCh37-hg19 and the chimpanzee reference sequence PanTro5 were retrieved from ENSEMBL [43]. RI loci for the different databases were identified in those tracks for information on genes containing RI.

Four human (All Genes, genes with RT-DB insertions, genes with AMH-specific vs chimp insertions and genes with AMH-specific vs HN/HD insertions) and three chimp gene-tracks (All Genes, genes with RT-DB Chimp insertions and genes with Chimp-specific RIs) were thus produced and compared for gene proportions and number of annotated transcripts. Proportion of RI occurred in genes were compared between the tracks and tested with Fisher and binomial tests in R.

The tracks were divided in series containing the number of annotated transcripts for each gene, which were then compared between each other and tested with Wilcoxon and Kolmogorov-Smirnov tests in R.

Spearman’s non-parametric correlation tests between gene length, number of transcripts and number of RT-DB insertions (two at a time) were also performed, followed by a partial correlation test between number of transcripts and number of insertions in function of gene length. These tests were executed (in R, functions cor.test and pcor.test respectively; parameter method = “spearman” in both cases) in order to exclude biases due to gene length and its impact on the random chance to observe features associated with it.

Functional annotation data on genes belonging to the aforementioned four human tracks were retrieved from DAVID Bionformatics Resources v6.8 [44]. For general preferential expression information, nomenclature of tissues belonging to cohesive histological complexes was merged under the categories “Brain”, “Testis”, “Epithelium”, “Placenta”, “Uterus”, “Lung”, “Liver”, “Lymph”, “Kidney”, “Eye”, “Muscle”, “Blood”, “Colon” and “Pancreas”. Only tissues individually called for preferential expression by at least 5% of all human genes were selected for the comparison.

For genes preferentially expressed in the brain, the categories were unpacked into “Brain (general)”, “Undifferentiated Neurons”, “Cerebellum”, “Amygdala”, “Hippocampus”, “Peripheral Nervous System”, “Thalamus”, “Cajal-Retzius Cells”, “Cortex”, “Pituitary”, “Hypothalamus”, “Caudate Nucleus”,“Dendritic Cells”, “Substantia Nigra”, “Subthalamic Nucleus”, “Corpus Callosum”. In this case, only tissues individually called for preferential expression by at least 0.5% of all human genes were selected for the comparison.

Tissue-by-tissue comparisons were tested using Fisher and binomial tests in R.

The lengths of genes displaying preferential expression in the different tissue types were also compared via a pairwise Wilcoxon test (pairwise.wilcox.test function in R with parameter p.adjust.method = “bonferroni”). This generated a matrix of *p*-values (one for each possible pairwise comparison), which was then inverted (1 - p-value) and used as a matrix of distances for a cluster analysis (hclust function in R, parameter method = “average”). A boxplot highlighting the length of the genes preferentially expressed in the various tissues and a dendrogram showing how the categories clustered in function of gene length were then plotted in R.

### GO functional analysis

To identify the gene-ontology category of the genes containing AMH-specific RI, both vs chimp and HN/HD, we used ToppCluster [45], which allows the identification of biological programs using different gene sets to perform contrast and comparative analysis. ToppCluster was set with a false-discovery-rate (FDR) threshold of 0.05 and using “GO: biological process” annotation. The obtained matrix was used to compute the –log10 p-values to obtain significance scores for each functional term. Next, to reduce the redundancy within the GO terms, we used REVIGO [77] with parameters set to C = 0.7, similarity measure “SimRel” [78] and using the *Homo sapiens* database. The scatterplots showing the representation of clusters from multidimensional scaling of the semantic similarities of GO terms were obtained with R. We used these plot to identify GO terms related with similar biological functions and the associated genes were used as input for GENEMania (default parameter) [79]. The networks obtained from REVIGO were downloaded and visualized with Cytoscape [80].

### Case studies insertional loci annotation and functional inference

The three AMH-specific RI absent in both HN and HD that were identified as recent and displaying peculiar population distribution were manually characterized using the UCSC Genome Browser [81], including genomic insertional locus, conservation of the sequence among primates, RepeatMasker presence/absence of repetitive elements, gene- and transcript-annotation.

To identify splicing motifs at the level of the insertion in the gene SHTN-1 we used Human Splicing Finder (HSF 3.0) [46]. HSF 3.0 was interrogated with the sequence of the RI + 100 bp of flanking regions and with the reconstructed flanking without the RI itself. This was repeated for the whole intron where the insertion occurred and for the reconstructed intron lacking the specific RI, in order to assess its possible effect in splicing-alteration.

## Additional files

Additional file 1: Schematic representation of the methodology used for RI identification between the compared species. Letters correspond to the various steps of the procedure that are described indetail in METHODS. Examples of RI sequences identified and validated by our methodology are reported below the methodological scheme. For HD- and HN-specific insertions, the three sequences represent: empty (pre-insertional) site in the modern human reference GRCh37-hg19, 5′ and 3′ portions of the insertion with flankings assembled from the archaic species DNA. For Chimp- and AMH-specific RI, the sequences are: empty (pre-insertional) site in one species’ reference genome, insertion with flankings in the other species’ reference genome. In all sequences, the inserted retrotransposon is represented in blue, the poly-A tail in yellow, the TSDs flanking the insertion or the single copy of the pre-insertional Target Site in red. The black rectangles on the empty (pre-insertional) sites indicate the exact location where the element inserted. All insertions described as species-specific in this work present the aforementioned characteristics. (PDF 126 kb)

Additional file 2: List of all identified and validated Chimp-specific RIs. (XLS 195 kb)

Additional file 3: List of all identified and validated HN-specific RIs. (XLS 92 kb)

Additional file 4: List of all identified and validated HD-specific RIs. (XLS 63 kb)

Additional file 5: List of all identified and validated AMH-specific RIs vs chimp. (XLS 485 kb)

Additional file 6: List of all identified and validated AMH-specific RIs vs HN. (XLS 132 kb)

Additional file 7: List of all identified and validated AMH-specific RIs vs HD. (XLS 97 kb)

Additional file 8: List of all identified and validated AMH-specific RIs vs both HN and HD. (XLS 68 kb)

Additional file 9: HD-, HN- and AMH-specific RI distribution in present-day populations. A-B) Heatmaps of respectively HD- and HN-specific RI distribution in present-day populations. Each line of the maps represents a single insertion, intensity of the color from grey to green reflects the frequency in modern populations. Arrows indicate three insertions that were identified as shared between HN and HD, rectangles highlight insertions that are putatively introgressed in modern populations post Out-of-Africa. C) Simulated curve representing random sampling of AFR individuals for the identification and exclusion of polymorphic archaic-specific insertions. Red dotted lines indicate 95% confidence intervals. D) Heatmap of AMH-specific RI distribution in in present-day populations: each line of the map represents a single insertion, intensity of the color from grey to purple reflects the frequency in modern populations. E) Neighbour joining tree calculated by using AMH-specific RIs in present-day populations as phylogenetic markers. Branches in orange are from populations with African descent, blue for European descent, green for South-Asian descent, red for East-Asian descent and black for Native-American descent, as clustered by RI distribution. (PDF 229 kb)

Additional file 10: Distribution of putatively HD-specific RIs in present day human populations. Polymorphic RIs and their frequency in modern populations from the 1000 Genomes Project are listed on top, with non-polymorphic RIs listed underneath them. Colors from green to red highlight progressively increasing frequencies of presence. (XLS 41 kb)

Additional file 11: Distribution of putatively HN-specific RIs in present day human populations. Polymorphic RIs and their frequency in modern populations from the 1000 Genomes Project are listed on top, with non-polymorphic RIs listed underneath them. Colors from green to red highlight progressively increasing frequencies of presence. (XLS 53 kb)

Additional file 12: Distribution of AMH-specific RIs in present day human populations. TMRCAs calculated for each insertional locus are also reported. Colors from green to red highlight progressively increasing frequencies of presence. (XLS 374 kb)

Additional file 13: 3P-CLR estimate on all AMH-specific RIs autosomal insertional loci. All insertional loci are listed in order of number of SNPs present in the 200 kb window that are associated with post-Out-of-Africa selection. (XLS 71 kb)

Additional file 14: Chimp-specific RIs in genes. Proportion of ENSEMBL-annotated genes in the whole reference genome PanTro5 (grey), proportion of insertions that occurred in annotated genes for RT-DB Chimp insertions (yellow) and Chimp-specific RIs (blue). In each diagram, the darker color denotes the percentage of RIs inserted in genes vs RIs inserted in non-genic regions (lighter color). B) Proportion of genes per number of annotated transcripts for all ENSEMBL-annotated genes in the reference genome PanTro5 (black dotted line), for genes with RT-DB Chimp insertions (yellow lines) and for genes containing Chimp-specific RIs (blue bars). C) Table showing statistical significance of the differences between the series of Additional file 14B, calculated with Kolmogorov-Smirnov tests; white is for non-significant *p*-value, emerald-green is for *p*-value < 10^− 10^, dark-green is for *p*-values < 10^− 16^. (PDF 103 kb)

Additional file 15: Distribution and clustering of genes preferentially expressed in specific tissues based on their length. A) Boxplot depicting the length of all human genes grouped by preferential expression. The letters under each box represent statistical similarity of the series (p-values < 0.05): two boxes sharing the same letter are not statistically different while two that do not share a letter are. B) Dendrogram representing a cluster analysis performed on all human genes grouped by preferential expression, using p-values resulting from the pairwise Wilcoxon test comparison between all series of length of the genes as a matrix of distances. Green/gray rectangle groups tissues in which generally short genes are preferentially expressed, red/blue rectangle groups tissues in which generally long genes are preferentially expressed, yellow/purple rectangle groups tissues in which genes with intermediate lengths are preferentially expressed. (PDF 157 kb)

Additional file 16: ToppCluster analysis results for GO terms associated with genes containing AMH-specific RIs. Loci enriched for sapiens-specific GO terms are listed first, followed by loci enriched for Hominina-specific GO terms and, finally, by loci with non-specific GO-terms. (XLS 159 kb)

## Abbreviations

3P-CLR: Three Population Composite Likelihood Ratio
AMH: Anatomically Modern Humans
GO: Gene Ontology
HD: Denisovan hominid
HN: *Homo Neanderthalensis*
N-LTRr: Non-LTR retrotransposon
RI: Retrotransposon Insertion
TE: Transposable Element
TMRCA: Time to the Most Recent Common Ancestor
