# Supplementary material for: Impact of non-LTR retrotransposons in the differentiation and evolution of anatomically modern humans
Source: Mob DNA. 2018 Aug 15;9:28. doi: 10.1186/s13100-018-0133-4 (PMC6094920; doi:10.1186/s13100-018-0133-4)
Supplement: Supplementary file 1 — Schematic representation of the methodology used for RI identification between the compared species. Letters correspond to the various steps of the procedure that are described indetail in METHODS. Examples of RI sequences identified and validated by our methodology are reported below the methodological scheme. For HD- and HN-specific insertions, the three sequences represent: empty (pre-insertional) site in the modern human reference GRCh37-hg19, 5′ and 3′ portions of the insertion with flankings assembled from the archaic species DNA. For Chimp- and AMH-specific RI, the sequences are: empty (pre-insertional) site in one species’ reference genome, insertion with flankings in the other species’ reference genome. In all sequences, the inserted retrotransposon is represented in blue, the poly-A tail in yellow, the TSDs flanking the insertion or the single copy of the pre-insertional Target Site in red. The black rectangles on the empty (pre-insertional) sites indicate the exact location where the element inserted. All insertions described as species-specific in this work present the aforementioned characteristics. (PDF 126 kb) [file 13100_2018_133_MOESM1_ESM.pdf]

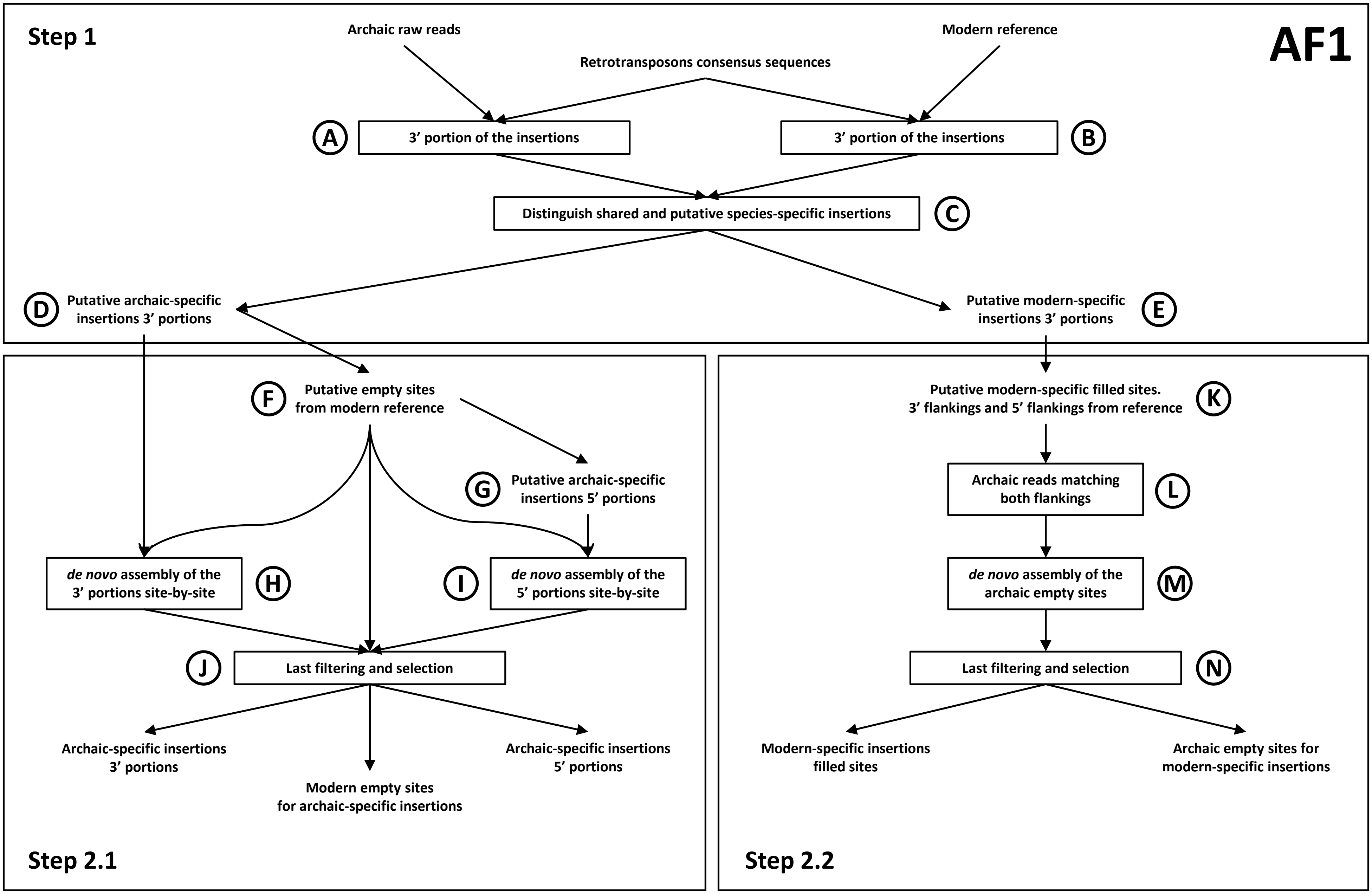

HD- and HN-specific insertions

>3:142605118-142605317_emptysite41038
gaacacatggccaaccatgaatttctacttgggcacatgacgcctctgagccattgattttctagggtcgtttaaaacttatggccatttaattacatttaaaacacaatcacgacatgaaaatcaagctgcttttatttttgtttcagagacttatttgacatttatgttaatagtatttcaaacttattttctatgac

>assembled_fs_5prime_emptysite41038
tcaaagtgtaaagtcacatcaacctgtccgcaatactcagaacacatggccaaccatgaatttctacttgggcacatgacgcctctgagccattgattttctagggtcgtttaaaacttatggccatttaattacatttaaaacacaatcacggccgggcgcggtggctcacgcctgtaatcccagcactttggga

>assembled_fs_3prime_emptysite41038
tgagccgagatcgcgccactgcactccaacctgggcgacagagcgagactctgtctcaaaaaaaaaaaaaaaaaaacacaatcacgatatgaaaatcaagctgcttttatttttgtttcagagacttatttgacatttat

• = AluY insertion 5' and 3' portions • = Poly-A tail • = Target Site Duplication

AMH-specific and Chimp-specific insertions

>assembled_empty_sites_filledsite1069
ctcagggagacctgtgtcagatttttatcctacagaactataagataatagatttatgttatttaagccactgatgttatggtaacttcttgcaggagcaataaaagactaataaacttactgaatcagaaactctatggtggggcccagacatctgtattttaacaagtcctccaagtgattctgatgcatactcaagt
tggagaaccactggcttagaactatgttgcttaggtaagttctttgccttccctgagcctcaacttcatcaactatcaaataggaaaaccatcac

>15:101452982-101453480_filledsite1069
gtcagggagacctgtgtcagatttttatcctacagaactataagataatagatttatgttatttaagccactgatgttatggtaacttcttgcaggagcaataaaagactaataaacttactgaatcagaaactctatggtggggcccagacatctgtattttttttttttttttgagacggagtctcgctctgtcgccc
aggctggagtgcagtggcgcgatctcggctcactgcaagctccgcctcccgggttcacgccattctcctgcctcagcctcccgagtagctgggactacaggcgcccgctgccacgcccggctaatttttgtatttttagtagagacggggtttcaccttgttagccaggatggtctcgatctcctgacctcgtgatccgcc
cgcctcggcctcccaaagtgctgggattacaggcgtgagccaccgcgcccggccgacatctgtattttaacaagtcctccaagtgattctgatgcatactcaagttggagaaccactggcttagaactatgttgcttaggtaagttctttgccttccctgagcctcaacttcatcaactatcaaataggaaaaccatcac

• = AluY insertion • = Poly-A tail (Poly-T) • = Target Site Duplication
