# Supplementary figures and images for: Impact of non-LTR retrotransposons in the differentiation and evolution of anatomically modern humans
Source: Mob DNA. 2018 Aug 15;9:28. doi: 10.1186/s13100-018-0133-4 (PMC6094920; doi:10.1186/s13100-018-0133-4)

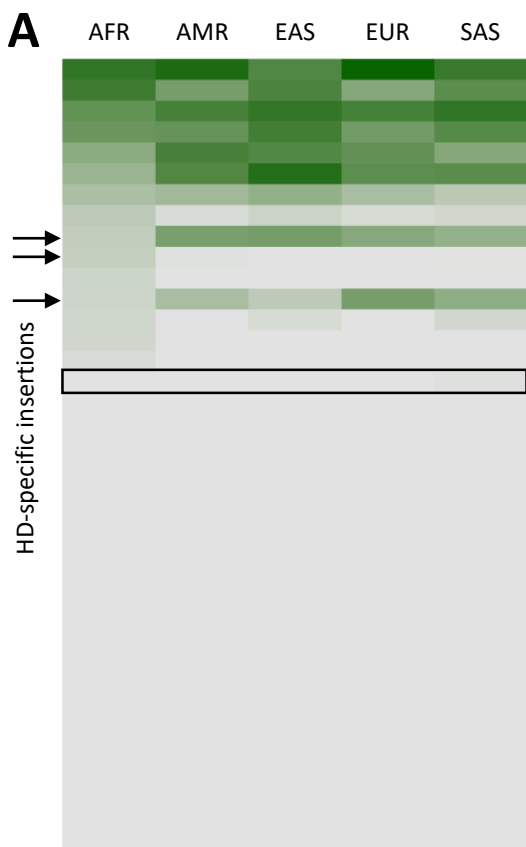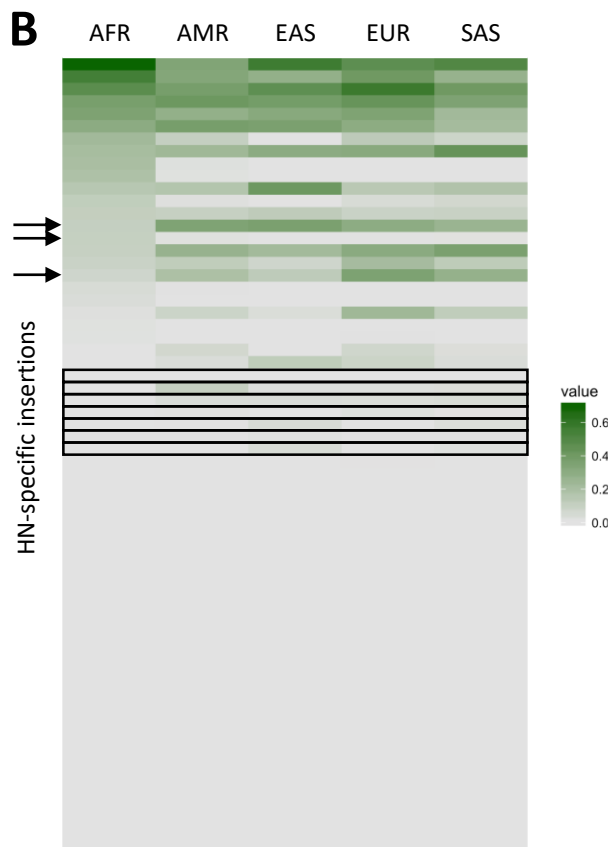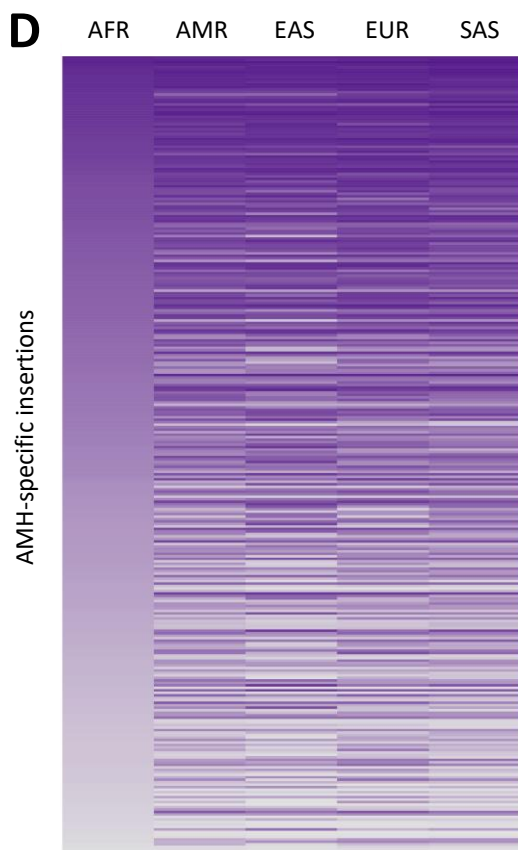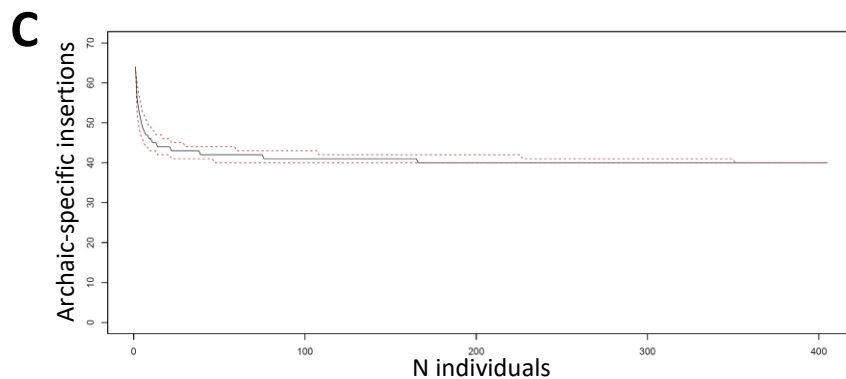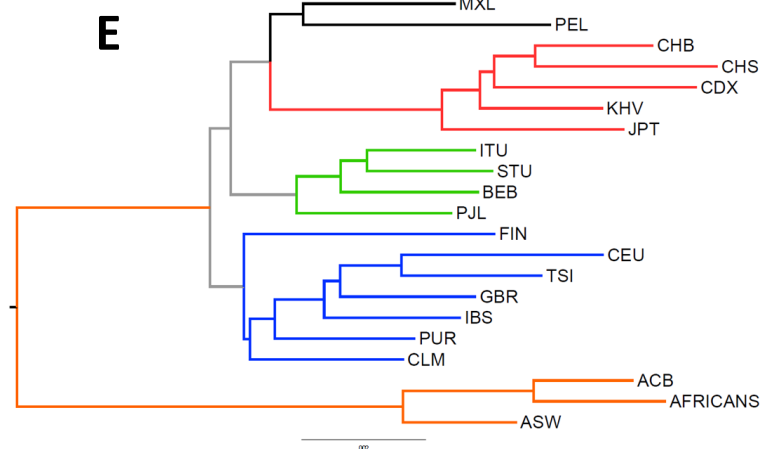

Supplement: Supplementary file 9 — HD-, HN- and AMH-specific RI distribution in present-day populations. A-B) Heatmaps of respectively HD- and HN-specific RI distribution in present-day populations. Each line of the maps represents a single insertion, intensity of the color from grey to green reflects the frequency in modern populations. Arrows indicate three insertions that were identified as shared between HN and HD, rectangles highlight insertions that are putatively introgressed in modern populations post Out-of-Africa. C) Simulated curve representing random sampling of AFR individuals for the identification and exclusion of polymorphic archaic-specific insertions. Red dotted lines indicate 95% confidence intervals. D) Heatmap of AMH-specific RI distribution in in present-day populations: each line of the map represents a single insertion, intensity of the color from grey to purple reflects the frequency in modern populations. E) Neighbour joining tree calculated by using AMH-specific RIs in present-day populations as phylogenetic markers. Branches in orange are from populations with African descent, blue for European descent, green for South-Asian descent, red for East-Asian descent and black for Native-American descent, as clustered by RI distribution. (PDF 229 kb) [file 13100_2018_133_MOESM9_ESM.pdf]

**A****PanTro5**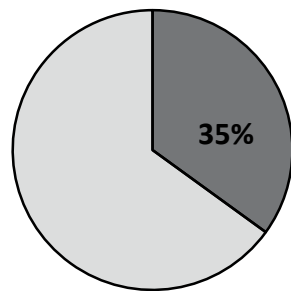**RT-DB Chimp**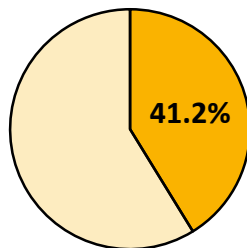

402,756 out of 976,979 RIs

**Chimp-specific**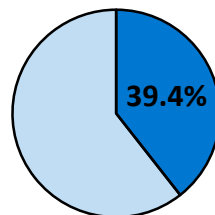

755 out of 1,917 RIs

**AF14****B**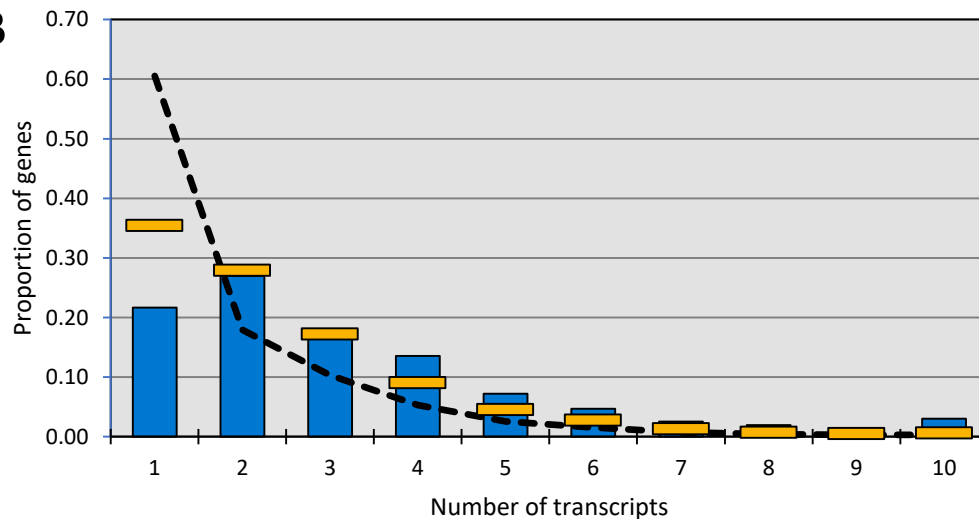**C**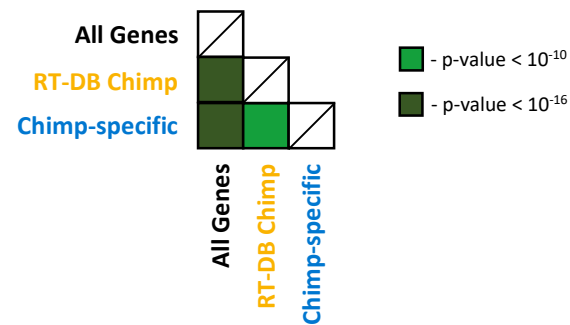

Supplement: Supplementary file 14 — Chimp-specific RIs in genes. Proportion of ENSEMBL-annotated genes in the whole reference genome PanTro5 (grey), proportion of insertions that occurred in annotated genes for RT-DB Chimp insertions (yellow) and Chimp-specific RIs (blue). In each diagram, the darker color denotes the percentage of RIs inserted in genes vs RIs inserted in non-genic regions (lighter color). B) Proportion of genes per number of annotated transcripts for all ENSEMBL-annotated genes in the reference genome PanTro5 (black dotted line), for genes with RT-DB Chimp insertions (yellow lines) and for genes containing Chimp-specific RIs (blue bars). C) Table showing statistical significance of the differences between the series of Additional file 14B, calculated with Kolmogorov-Smirnov tests; white is for non-significant p-value, emerald-green is for p-value < 10− 10, dark-green is for p-values < 10− 16. (PDF 103 kb) [file 13100_2018_133_MOESM14_ESM.pdf]

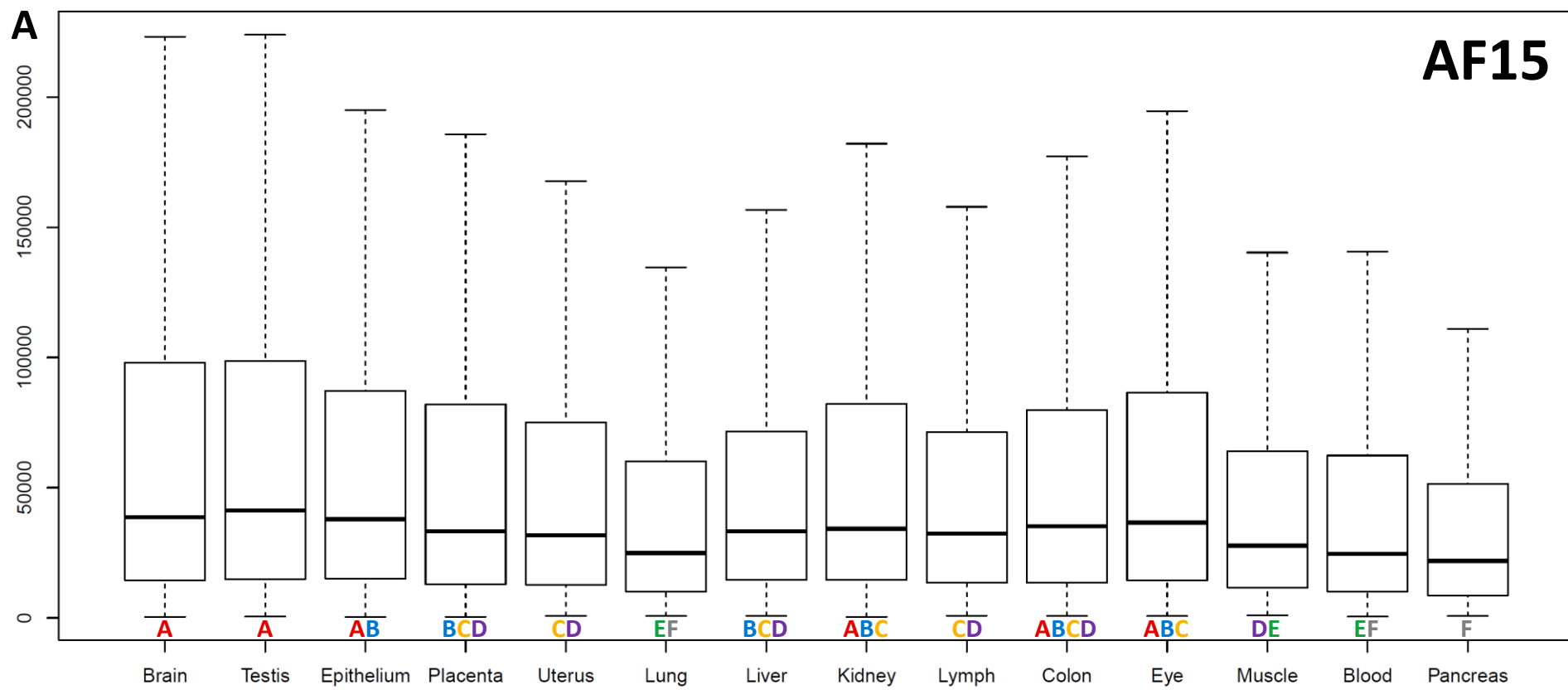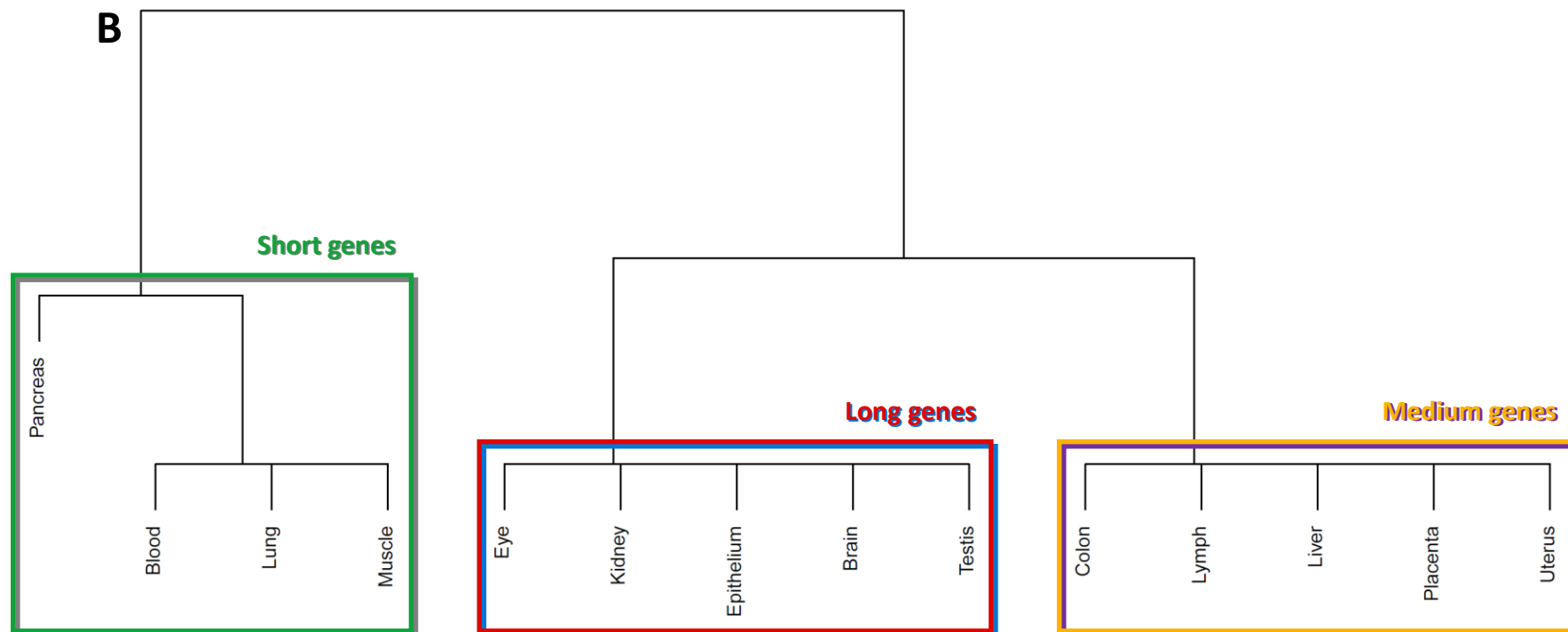

Supplement: Supplementary file 15 — Distribution and clustering of genes preferentially expressed in specific tissues based on their length. A) Boxplot depicting the length of all human genes grouped by preferential expression. The letters under each box represent statistical similarity of the series (p-values < 0.05): two boxes sharing the same letter are not statistically different while two that do not share a letter are. B) Dendrogram representing a cluster analysis performed on all human genes grouped by preferential expression, using p-values resulting from the pairwise Wilcoxon test comparison between all series of length of the genes as a matrix of distances. Green/gray rectangle groups tissues in which generally short genes are preferentially expressed, red/blue rectangle groups tissues in which generally long genes are preferentially expressed, yellow/purple rectangle groups tissues in which genes with intermediate lengths are preferentially expressed. (PDF 157 kb) [file 13100_2018_133_MOESM15_ESM.pdf]
